# The usefulness of plasma levels of mature and total adrenomedullin as biomarkers indicating the magnitude of surgical stress responses: A single-center, prospective, observational study

**Published:** 2021-05-14

**Authors:** Go Otao, Toyoaki Maruta, Tetsu Yonaha, Koji Igarashi, Sayaka Nagata, Kazuo Kitamura, Isao Tsuneyoshi

**Affiliations:** ^1^Department of Anesthesiology, Faculty of Medicine, University of Miyazaki, Miyazaki 889-1692, Japan; ^2^Research and Development Management, Bioscience Division, TOSOH Corporation, Ayase 252-1123, Japan; ^3^Circulatory and Body Fluid Regulation, Department of Internal Medicine, Faculty of Medicine, University of Miyazaki, Miyazaki 889-1692, Japan

**Keywords:** adrenomedullin, biomarkers, perioperative period, prospective study, sequential organ failure assessment score

## Abstract

**Background and Aim:**

Adrenomedullin (AM), a vasodilatory peptide, is known for its pleiotropic actions. AM levels are increased under inflammatory conditions such as sepsis and can be useful as a prognostic biomarker. However, there are only a few reports on the physiological actions of AM in the perioperative period. The aim of this single-center, prospective, and observational study was to investigate the changes in the plasma levels of mature AM (mAM) and total AM (tAM) observed during the perioperative period. In addition, we aimed to determine the association between each AM level and immune-inflammatory parameters to explore the usefulness of AM as a biomarker of the magnitude of surgical stress responses.

**Methods:**

The levels of both mAM and tAM, in addition to the levels of presepsin, interleukin-6, procalcitonin, white blood cell, and C-reactive protein, were measured in blood samples obtained during the perioperative period. Other laboratory data, including sequential organ failure assessment (SOFA) and acute physiology and chronic health evaluation (APACHE) II scores, were obtained from individual clinical records. Correlations between each AM and clinical parameters were determined using Spearman’s rank correlation. *P*<0.05 were considered statistically significant.

**Results:**

One hundred and twenty-three perioperative patients scheduled for three types of surgical procedures, including cardiopulmonary bypass surgery, abdominal surgery, and cervical laminoplasty, were included in this study. There was a moderate to strong correlation between each AM and immune-inflammatory parameters, SOFA score, and APACHE II score, as related to surgical trauma. Specifically, the strongest correlation was observed between each AM and SOFA score.

**Conclusions:**

These findings suggest that plasma AM levels may represent the most important inflammatory mediators that are evident in surgical stress responses.

**Relevance for patients:**

Since the levels of both tAM and mAM show the same trend, mAM and tAM may be equally used as biomarkers for the evaluation of the physiological status of surgical patients.

**Trial Registration:**

This observational study was retrospectively registered with Japanese Clinical Trial Registry “UMIN-CTR” on March 19, 2018, and was given a trial ID number UMIN000031792.

## 1. Introduction

Perioperative outcomes have shifted from post-operative mortality to long-term mortality, even after hospital discharge [1,2], because perioperative medicine is significantly associated with long-term survival. Post-operative complications are the most important predictor of long-term mortality; thus the stratifying pre-operative risk and guiding appropriate perioperative care has acquired critical importance to prevent post-operative adverse events. Perioperative biomarkers may improve the predictive precision of clinical risk scores used to guide perioperative management [1]. The development of useful perioperative biomarkers should depend on prospective clinical data that enable the identification of high-risk patients as well as the translational understanding of pathophysiological mechanisms underlying post-operative organ dysfunctions [2].

Adrenomedullin (AM) is a potent vasodilator peptide originally identified in the tissue extract of human pheochromocytoma [3]. However, in the present day, AM is known to be produced in various organs and tissues [4] and has various physiological actions on the cardiovascular, renal, and central nervous systems, including the regulation of blood pressure and vascular tone, increase in cardiac output, diuresis and natriuresis, inhibition of aldosterone secretion, and suppression of fluid intake [3-8]. Blood levels of AM increase in patients with heart failure, myocardial infarction, pulmonary hypertension, systemic inflammatory response syndrome, sepsis, inflammatory bowel diseases, or renal failure [4,9-12]. In particular, in sepsis, the blood level of AM is associated with sequential organ failure assessment (SOFA) or acute physiology and chronic health evaluation (APACHE) II scores, especially at high scores, and therefore, AM can be useful as a prognostic biomarker [13-20]. Furthermore, on the basis of these pharmacological properties, AM is considered a potential therapeutic target, and clinical trials have been conducted to demonstrate the efficacy of AM in the treatment of acute myocardial infarction, primary pulmonary hypertension, peripheral artery disease (arteriosclerosis obliterans or Buerger’s disease), and ulcerative colitis [4]. In contrast, the monoclonal antibody against AM, adrecizumab, has been under a clinical trial (AdrenOSS-2) for its use to treat patients with septic shock [21]. However, there are only a few reports on the role of AM during the perioperative period, where it can occasionally lead to a variety of profound physiological alterations characterized by changes in hemodynamics and endocrine and immune functions dependent on surgical stress.

In this study, we aimed to determine whether the magnitude of surgical stress exerted on patients affected the release of AM, including the bioactive mature form (mature AM [mAM]), the intermediate form of AM [iAM], and the total mixture of mAM and iAM (tAM). In addition, we examined the correlation between AM levels and immune-inflammatory parameters, such as the SOFA score and APACHE II score, in these patients to explore the usefulness of AM as a biomarker of the surgical stress response.

## 2. Materials and Methods

### 2.1. Ethics and study design

This study was conducted in accordance with the principles of the Declaration of Helsinki. The study was approved by the institutional Ethical Committee for Human Studies (Ref: xxxx) on November 21, 2014, and retrospectively registered with the Japanese Clinical Trial Registry “UMIN-CTR” (Ref: UMIN000031792) [22]. Written informed consent was obtained from all patients (age ≥20 years). The Strengthening the Reporting of Observational Studies in Epidemiology guidelines were followed in the preparation of this manuscript.

We studied elective surgical patients (American Society of Anesthesiologists physical status I-III) who underwent nine kinds of surgeries: cardiac surgery, aortic arch replacement surgery, coronary artery bypass grafting (CABG) surgery, abdominal aortic replacement surgery, hepatectomy, pylorus-preserving pancreatoduodenectomy (PPPD), esophagectomy, free jejunal grafting with otolaryngological surgery, and cervical laminoplasty. These surgeries were chosen according to three requirements: Surgeries with varying levels of operative stress, arterial catheter insertion, and post-operative intensive care unit (ICU) admissions. Arterial catheter insertion was useful in alleviating physical pain from frequent blood drawing. In the ICU, patients were taken for copious medical checks and detailed progress tables were recorded, which were useful in calculating SOFA and APACHE II scores. While cervical laminoplasty was selected as minimal stress surgery, other selected surgeries were categorized into two types: Cardiopulmonary bypass (CPB) surgeries and major abdominal surgeries. CPB surgeries included cardiac, aortic arch replacement, and CABG surgeries, whereas major abdominal surgeries included abdominal aortic replacement surgery, hepatectomy, PPPD, esophagectomy, and free jejunal grafting with otolaryngological surgery. Exclusion criteria included age <19 years, history of emergency surgery, the requirement of hemodialysis, pregnancy, and lack of informed consent.

### 2.2. Measurements and data collection

To measure serial AM, presepsin, and interleukin-6 (IL-6) levels, arterial blood samples were obtained through the radial artery catheter at the following intervals: (a) Immediately before surgery, (b) at the end of the surgery, (c) on post-operative day 1 (POD1), and (d) at POD2; thus, four samples and maximum four measured values were obtained per patient. Seven milliliters of blood were collected into tubes with 1.0 mg/ml EDTA-2Na at each interval. Plasma was obtained by centrifugation at 1710 × g for 10 min at 4°C and stored at −80°C until analysis. AM was measured from each plasma sample, while presepsin and IL-6 levels were measured from the residuals.

**Figure 1:**
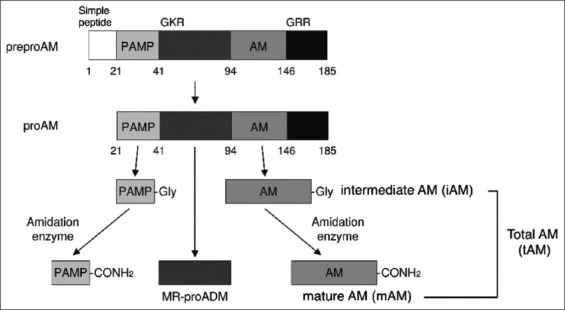
Schematic representation of the processing of AM from preproAM. AM is produced from AM precursor in two steps through an enzymatic reaction. First, AM precursor consisting of 185 amino acids, called preproAM, is converted to glycine-extended AM, a 53-amino acid peptide of an inactive intermediate form of AM (iAM). Subsequently, iAM is converted to bioactive mature AM (mAM), a 52-amino acid peptide with a C-terminal amide structure, by enzymatic amidation. AM: Adrenomedullin; GKR: Glycine-lysine-arginine; GRR: Glycine-arginine-arginine; PAMP: Proadrenomedullin N-terminal 20 peptide; MR-proADM: Midregional pro-adrenomedullin; iAM: Intermediate form of adrenomedullin; mAM: Mature adrenomedullin; tAM: Total adrenomedullin.

AM is produced from AM precursor in two steps through an enzymatic reaction (Figure 1). First, an AM precursor consisting of 185 amino acids, called preproadrenomedullin, is converted to glycine-extended AM, a 53-amino acid peptide of an inactive iAM. Subsequently, iAM is converted to bioactive mAM, a 52-amino acid peptide with a C-terminal amide structure, by enzymatic amidation [23,24]. Although both iAM and mAM forms circulate in the bloodstream, over 85% of total plasma AM is in the inactive iAM form. Comparative radioimmunoassays used in previous studies could not distinguish iAM and mAM; hence, AM sometimes indicates tAM (iAM + mAM). Plasma levels of both mAM and tAM were measured by a specific fluorescence immunoassay (Tosoh Corporation, Tokyo, Japan) with two independent antibodies: for the tAM assay, one that binds to the ringed structure (amino acid 12–25) and the other to the middle region (amino acid 25–36) between the ring and the C-terminal portions; and for the mAM assay, one that binds to the ringed structure (amino acid 12–25) and the other to the C-terminus (amino acid 46–52), as previously described [25,26]. Plasma presepsin levels were measured with a rapid chemiluminescent enzyme immunoassay using a PATHFAST immunoanalyzer (Mitsubishi Chemical Medience, Tokyo, Japan). IL-6 was measured using the human IL6 Single-Analyte ELISA Kit (QIAGEN, Maryland, USA). The surgical stress score (SSS) is part of the estimation of physiologic ability and surgical stress, which comprises the pre-operative risk score, SSS, and the comprehensive risk score [27]. In addition, we obtained data on procalcitonin (PCT), white blood cell (WBC), and C-reactive protein (CRP) levels, which were measured from serum samples at the central laboratory.

SOFA and APACHE II scores were also assessed as clinical parameters indicating the general condition. These scores were calculated (a) preoperatively, (b) on the day of surgery, (c) on POD1, and (d) on POD2. When arterial blood gas data were not available, PaO_2_ was derived from SpO_2_ by Hill’s equation, and pH and HCO_3_ were calculated as 0 points each. In intubated patients, a verbal response on the Glasgow Coma Scale was calculated as 1 point (VT). In the absence of rectal or other core temperature values, axillary temperature plus 1°C was used as an alternative. Pre-operative and intraoperative data for the SOFA and APACHE II scores were collected by study staff, whereas the data during ICU stay were routinely collected by ICU staff.

### 2.3. Statistical analysis

Statistical analysis was performed using JMP 11 (SAS Institute Inc., Cary, NC, USA) and MedCalc 17 (MedCalc Software Bvba, Ostend, Belgium). Data are expressed as mean (SD), median (IQR), or numbers. Normally distributed data were analyzed using one-way analysis of variance (ANOVA) with post-hoc mean comparison by the Tukey–Kramer honestly significant difference (HSD) test. Correlations were determined using Spearman’s rank correlation coefficient. Spearman’s rank correlation was performed by pairing two parameters within each time point (a, b, c, or d). *P*<0.05 was considered statistically significant.

## 3. Results

**Figure 2:**
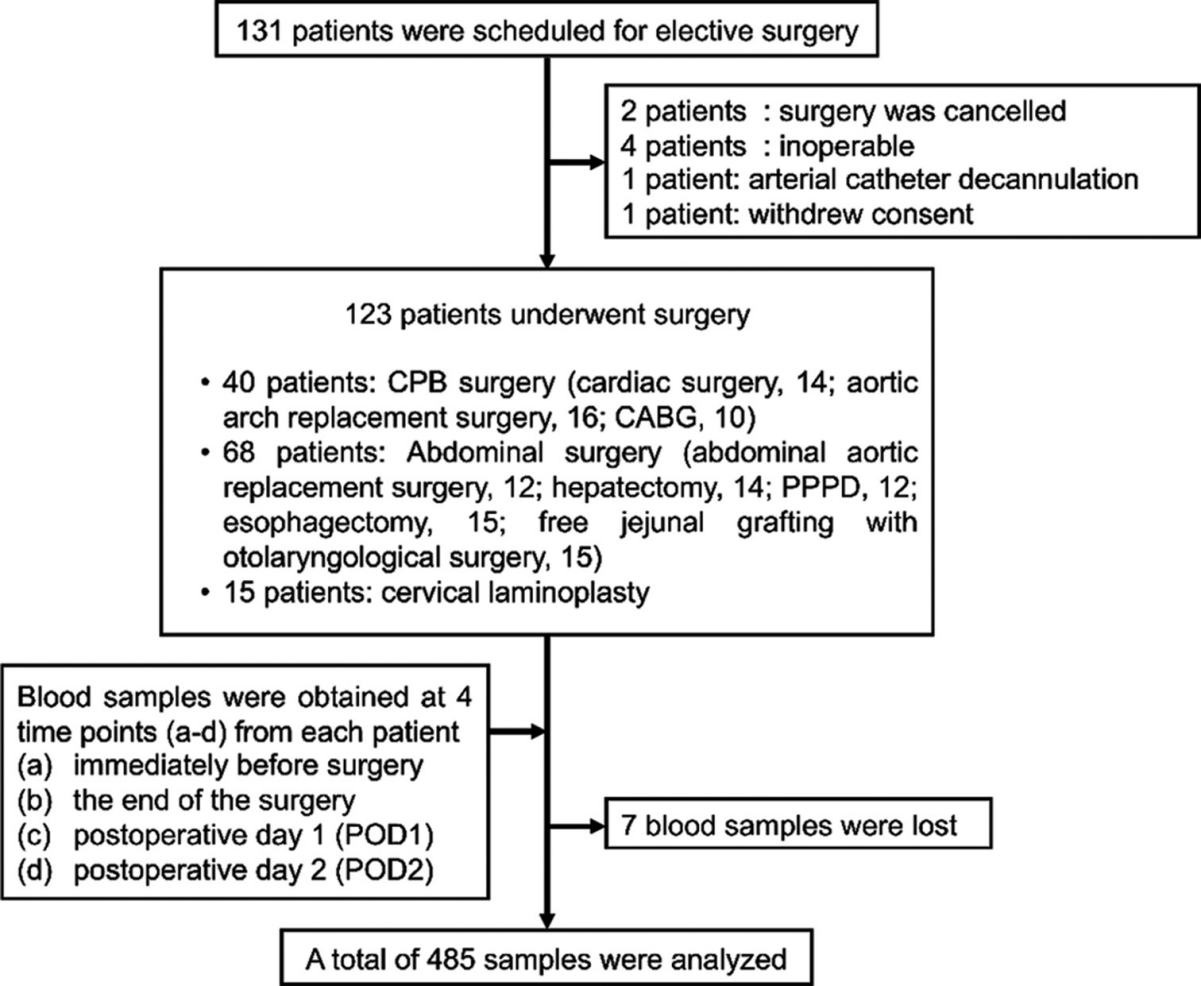
Flowchart of patient enrollment and sample collection. In total, 131 patients were recruited from December 2014 to October 2016. In two patients, surgeries were canceled; four patients were inoperable; in one patient, a blood sample could not be obtained due to accidental arterial catheter decannulation; and one patient withdrew consent. Finally, 123 patients who were scheduled for nine kinds of surgeries were enrolled (cardiac surgery, 14; aortic arch replacement surgery, 16; CABG, 10; abdominal aortic replacement surgery, 12; hepatectomy, 14; PPPD, 12; esophagectomy, 15; free jejunal grafting with otolaryngological surgery, 15; cervical laminoplasty, 15). To measure serial AM, presepsin, and interleukin-6 (IL-6) levels, blood samples were obtained at the following 4-time points: (a) Immediately before the surgery, (b) at the end of the surgery, (c) on post-operative day 1 (POD1), and (d) on post-operative day 2 (POD2). Blood samples from 123 patients at 7-time points were lost, and thus, finally, a total of 485 analyzable samples were obtained. CPB: Cardiopulmonary bypass; CABG: Coronary artery bypass grafting; PPPD: Pylorus-preserving pancreatoduodenectomy.

A flowchart of patient enrollment is shown in Figure 2. In total, 131 patients were recruited from December 2014 to October 2016. For two patients, surgeries were canceled; four patients were inoperable; for one patient, a blood sample could not be obtained due to accidental arterial catheter decannulation; and one patient withdrew consent. Finally, 123 patients in the three surgery groups were enrolled (CPB surgery, 40; abdominal surgery, 68; cervical laminoplasty, 15). Seven blood samples were lost, and a total of 485 analyzable samples were obtained.

**Figure 3:**
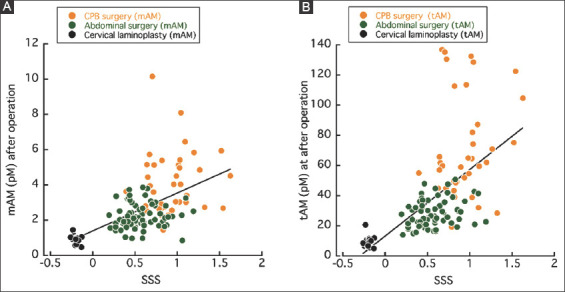
The relationships between SSS and mAM (A) or tAM (B). y = 2.15x + 1.39 (y = mAM and x = SSS); R2 = 0.32 and y = 44.09x + 13.24 (y = tAM and x = SSS); R2 = 0.36, respectively. CPB: Cardiopulmonary bypass; SSS: Surgical stress score; mAM: Mature adrenomedullin; pM: Picomolar; tAM: Total adrenomedullin.

Patient characteristics are shown in Table 1. Figure 3A and B shows the relationship between each AM and SSS and indicates that patients with a wide range of surgical stress were enrolled in the present study.

**Table 1:** Data characteristics of patients stratified by surgery

	Total	CPB surgery	Abdominal surgery	Cervical laminoplasty	*P*-value
n	123	40	68	15	
Male/Female	80/43	20/20	50/18	10/5	0.05
Age (years)	70 (63–77)	71 (67–79)	69 (63–75)	64 (50–72)	<0.01
Height (cm)	160 (154–165)	160 (148–162)	162 (156–167)	161 (151–164)	0.01
Weight (kg)	58 (51-68)	61 (48–65)	59 (51–67)	64 (51–71)	<0.01
Anesthetic technique					<0.01
Volatile	48	14	19	15	
TIVA	26	23	3	0	
Volatile+TIVA	4	3	1	0	
Volatile+Epidural	33	0	33	0	
TIVA+Epidural	10	0	10	0	
Volatile+TIVA+Epidural	2	0	2	0	
Surgical time (min)	417 (340–564)	416 (352–445)	535 (372–673)	191 (167–213)	<0.01
Anesthesia time (min)	519 (427–677)	529 (457–541)	623 (465–727)	272 (236–294)	<0.01
Blood transfusion (ml)	1050 (0–2177)	2210 (1958–2606)	555 (0–1364)	0	<0.01
Infusion (ml)	4235 (2795–5298)	4600 (2925–5033)	4819 (3683–5550)	1850 (1525–2075)	<0.01
Blood loss (g)	1110 (330–2246)	2200 (2088–2875)	665 (345–1383)	50 (50–110)	<0.01
Urine (ml)	1320 (800–2260)	1500 (1284–2485)	1300 (798–2273)	780 (550–1085)	<0.01
Balance (ml)	2295 (1320–3308)	2164 (990–2676)	3019 (2265–4053)	810 (560–1360)	<0.01

The results are expressed as median (IQR) or numbers of patients. CPB: Cardiopulmonary bypass; TIVA: Total intravenous anesthesia; IQR: Interquartile range. *P-value from multigroup comparisons using nonparametric Kruskal–Wallis or Fisher’s exact test.

**Figure 4:**
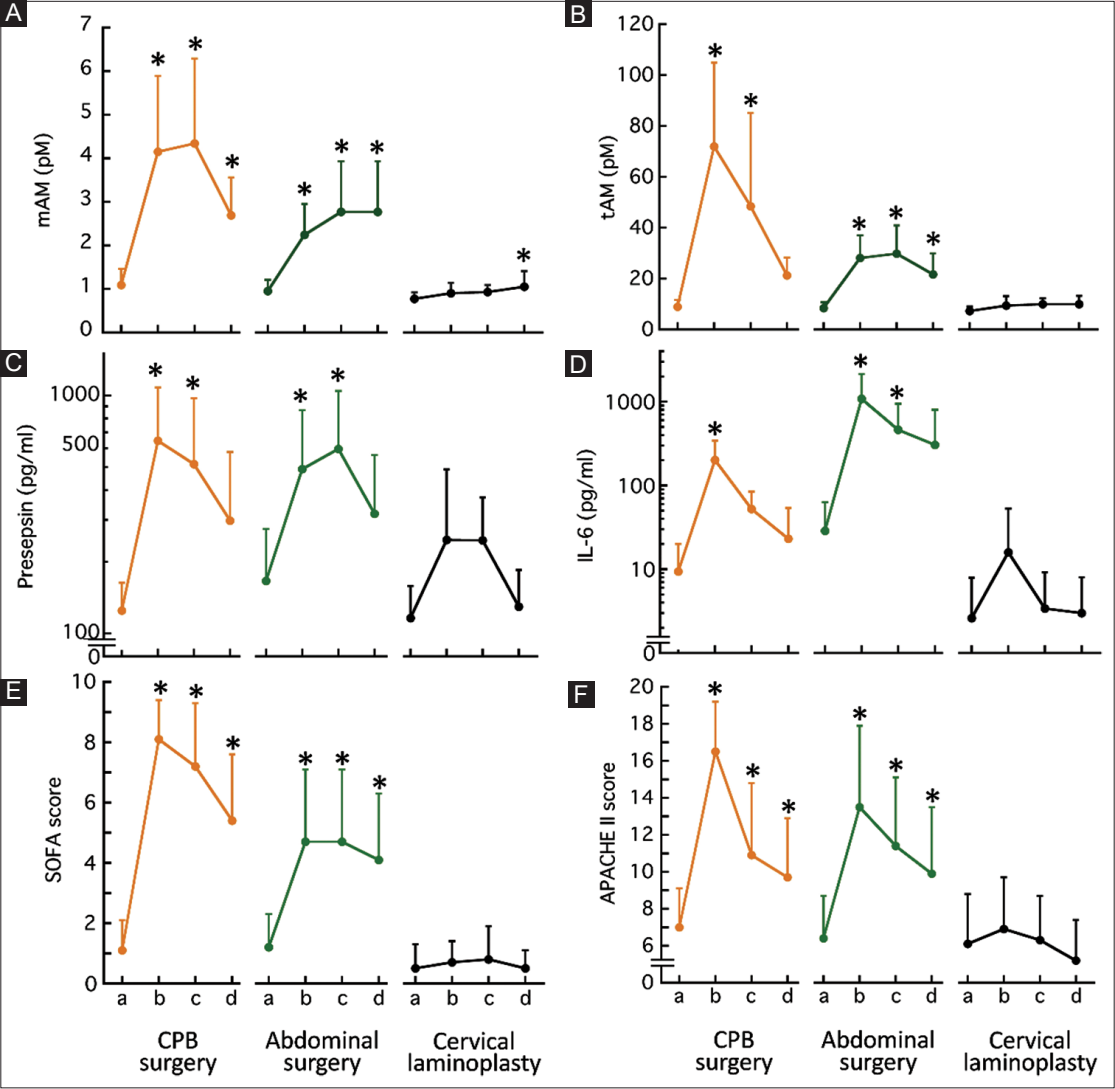
Time courses of mAM (A), tAM (B), presepsin (C), IL-6 (D), SOFA score (E), and APACHE II score (F) in each surgery group. The levels of AM, presepsin and IL-6 were measured from arterial blood samples obtained (a) just before surgery, (b) at the end of the surgery, (c) at post-operative day 1 (POD1), and (d) at post-operative day 2 (POD2). Within each surgery group (CPB, abdominal, and cervical laminoplasty), the time courses of tAM and mAM were similar. The data are presented as means ± SD. *P<0.05 compared with pre-operative value within each group. mAM: Mature form of adrenomedullin; pM: Picomolar; tAM: Total adrenomedullin; IL-6: Interleukin-6; SOFA: Sequential organ failure assessment; APACHE: Acute physiology and chronic health evaluation; CPB: Cardiopulmonary bypass.

Figure 4A and B shows the serial mAM and tAM levels in each surgery group. Basal (pre-operative) mAM and tAM levels did not differ between the groups. Within each surgery group, the changes in mAM and tAM levels were analogous, and the peak levels of mAM and tAM in CPB and abdominal surgery occurred at the end of surgery or on POD1. The least changes in perioperative AM levels occurred in cervical laminoplasty cases. In Table 2, the correlations between each AM level and immune-inflammatory parameters, SOFA score, and APACHE score are shown. mAM level was moderately to strongly correlated with values of presepsin, IL-6, PCT, WBC, CRP, SOFA score, and APACHE II score (Spearman’s rho = 0.60, 0.47, 0.42, 0.58, 0.49, 0.72, and 0.58, respectively). tAM level was also moderately to strongly correlated with values of presepsin, IL-6, PCT, WBC, CRP, SOFA score, and APACHE II score (Spearman’s rho = 0.66, 0.60, 0.46, 0.51, 0.44, 0.72, and 0.61, respectively). The serial presepsin and IL-6 levels and SOFA and APACHE II scores in each surgery are shown in Figure 4C,4D,4E, and 4F, respectively. The peak scores of SOFA and APACHE II were obtained at the end of surgery or on POD1 in the same manner as AM levels. Among the parameters, the SOFA score was the most strongly correlated one with mAM or tAM levels (Table 2).

**Table 2:** Correlation analyses between each AM and immuneinflammatory parameters, the SOFA score, and APACHE II score

	The correlation analysis of mAM		The correlation analysis of tAM	
	
Parameters	Spearman’s Rho (95% CI)	*P*-value	Spearman’s Rho (95% CI)	*P*-value
Presepsin	0.603	<0.0001	0.656	<0.0001
IL-6	0.471	<0.0001	0.603	<0.0001
PCT	0.423	0.0001	0.464	<0.0001
WBC	0.584	<0.0001	0.512	<0.0001
CRP	0.492	<0.0001	0.437	<0.0001
SOFA	0.723	<0.0001	0.724	<0.0001
APACHE II	0.580	<0.0001	0.607	<0.0001

Correlation analyses between each AM and immune-inflammatory parameters, SOFA score, and APACHE II score. Presepsin and IL-6 levels were measured, and the scores were calculated. PCT, WBC, and CRP values were measured as part of daily clinical practice. Presepsin and IL-6 levels had missing data due to loss of residuals, and other laboratory parameters had missing data when not measured clinically. AM: Adrenomedullin; CI: Confidence interval; SOFA: Sequential organ failure assessment; APACHE: Acute physiology and chronic health evaluation; IL-6: Interleukin-6; PCT: Procalcitonin; WBC: White blood cell; CRP: C-Reactive protein.

## 4. Discussion

In the current study, we showed that (1) the peak AM levels in CPB surgeries were higher than those in major abdominal surgeries and less traumatic surgical interventions such as cervical laminoplasty; (2) both mAM and tAM levels were highly correlated with immune-inflammatory parameters, including WBC, CRP, presepsin, IL-6, and PCT, and especially with the scoring systems (SOFA and APACHE II) widely used in ICUs globally to assess organ dysfunction and severity of the physical condition; and (3) changes in the levels of mAM and tAM were similar in each perioperative period. These results suggest that both mAM and tAM may be equally used as biomarkers for measuring the magnitude of the surgical stress response.

Our present study revealed that operative stress widely differed between enrolled surgeries, and the elevated levels of AM caused by operation differed among different types of surgery. Few reports on AM in CPB and major abdominal surgeries exist [28-31]. In our study, CPB surgery and major abdominal surgery with high SSS had high peak AM levels, which is consistent with previous reports [28-31], whereas cervical laminoplasty with low SSS did not have significantly elevated tAM and mAM levels. A significant correlation between the increment in AM levels and aortic cross-clamp time has been reported [29]. Nishikimi *et al*. suggested that because the main source of plasma AM was the endothelium, the elevated AM levels during CPB could have been produced by the vascular wall, stimulated by activated monocytes, lymphocytes, and leukocytes through cytokines [30].

Other authors have reported similar findings. It was reported that the elevation of perioperative midregional proadrenomedullin (MR-proADM) levels was associated with the intensity of surgical trauma in open vascular surgery [32]. Furthermore, Schoe *et al*. reported that MR-proADM was a good predictor of hospital mortality in elective cardiac surgery [33]. However, as described in Figure 1, MR-proADM is an inactive byproduct generated during mAM production. Therefore, the present work is the first description of the changes in the secretion of bioactive mAM in various types of surgical procedures to provide evidence that the measurements of tAM could reflect the functional status of mAM *in vivo*.

In our study, AM level was associated with the levels of immune-inflammatory parameters, including presepsin, IL-6, PCT, WBC, and CRP. In previous studies, the plasma AM level demonstrated utility as a diagnostic and prognostic biomarker in patients with sepsis [13-18], similar to presepsin and PCT levels [34,35]. Although the cut-off point of the presepsin level in sepsis was 500 pg/ml, the post-operative presepsin level was elevated above this cut-off point in our study. Our findings suggest that presepsin levels increase under non-infectious, high-inflammatory conditions. IL-6 levels are correlated with AM levels in major abdominal surgery [30] and peripheral arterial occlusive disease [36]. As stated above, cytokines are released during systematic inflammation due to surgical stress, infection, and inflammation of the blood vessel itself, and inflammatory cytokines such as IL and tumor necrosis factor stimulate AM production and secretion from vascular smooth muscle cells [37,38].

In a study of 352 ICU patients, an initial SOFA score of up to 9 or >11 predicted mortality rates of <33% and 95%, respectively [39]. The highest SOFA score of 10 correlated with a mortality rate of 40%, while those higher than 11 were associated with mortality rates >80% [39]. Godinjak *et al*. demonstrated that in 174 medical ICU patients, the APACHE II score was a statistically significant predictive marker of fatal outcomes in patients (area under the receiver operator curve, 0.92; 95% confidence interval, 0.87–0.97, *P*=0.001) [40]. The cut-off value of the APACHE II score was 27.5, and the sensitivity and specificity were 74.5% and 93.3%, respectively [40]. It was also shown that SOFA and APACHE II scores demonstrated powerful discrimination or calibration of morbidity and mortality in cardiac surgical patients [41]. In particular, the SOFA score is now included in the new clinical criteria (Sepsis-3) for defining sepsis [42]. In the present study, the AM level was more strongly correlated to SOFA and APACHE II scores than the laboratory data. These findings suggest that perioperative AM levels could reflect the prognosis of these surgical patients. More information is urgently needed on the outcome of patients with elevated AM levels due to surgical stress.

Recently, Weber *et al*. reported a new assay that was developed to reliably measure the bioactive mAM (referred to as bio-ADM) [43]. This bio-ADM assay uses two monoclonal antibodies against amino acid 21–32 and 42–52. The limits of detection and quantitation were 3 and 11 pg/ml (=0.50 and 1.83 pM), respectively. Bio-ADM levels in patients with sepsis are more strongly associated with clinical outcomes than MR-proADM concentrations [16]. Whilst, in our mAM assay, the limits of detection and quantitation were determined to be 0.133 and 0.085 pM (=0.80 and 0.51 pg/ml), respectively, according to the Clinical and Laboratory Standards Institute protocols. Intra- and inter-assay coefficients of variation of this assay were 1.8% and 5.1%, respectively [26]. The antibodies used in our mAM assay and the antibodies used in the bio-ADM assay recognize locations that are in close proximity to one another. However, the clear-cut influence of complement factor H in our assay has not been reported. Thus, we consider that this assay is as reliable as the bio-ADM assay reported by Weber *et al*. unless complement factor H concentration is especially high.

In acute heart failure, bio-ADM performs as a suitable biomarker for the severity of congestion and 1-year mortality [44]. Furthermore, the correlation of the bio-ADM plasma levels on ICU admission with the requirement and quantity of vasopressors and mortality in sepsis is well established [13,14,16,45]. Kim *et al*. reported that bio-ADM concentration is significantly higher among patients with septic shock, vasopressor use, and in non-survivors than in patients with solitary sepsis, no vasopressor use, and in survivors. Further, bio-ADM concentration and SOFA score equally in predicting 30-day mortality [45]. Marino *et al*. reported that patients with sepsis who require vasopressors on admission have significantly higher bio-ADM levels on admission than those who do not and that admission ADM levels are strongly associated with 28-day mortality [16]. Simon *et al*. reported that during the hospital stays, patients on vasopressor therapy in the ICU exhibit significantly higher bio-ADM levels after 16 h of sepsis than patients without any vasopressor therapy [14]. Caironi *et al*. reported that the inotropic score over the first 7 days of treatment for septic patients alive at that time is greater in those with higher levels of bio-ADM at day 1 compared with those with lower levels, and bio-ADM concentrations on day 1 are independently associated with 90-day mortality [13]. These findings suggest that the mature bioactive AM (tAM or bio-ADM) is recognized as a biomarker of organ failure, similar to the conventional total AM (tAM). Our present study findings also support evidence that mAM reflects the degree of organ damage, even during the perioperative period.

### 4.1. Limitations

Our study has several limitations. Very few parameters returned to baseline during our short observational period (up to POD2); thus, a longer observation period is needed. The effect of complement factor H on the mAM assay used in our study is not yet known. Anesthetic management depended on the anesthesiologist. Anesthetic techniques might affect the release of AM. We could not provide any evidence of the role of AM in the pathogenesis of perioperative organ failure. This was a single-center study. Therefore, large multi-center studies are essential to determine if similar results can be detected in the settings of other surgical procedures and to clarify the clinical significance of long-term follow-up examinations, to elucidate the relationship between AM levels and surgical outcomes.

## 5. Conclusions

This observational study supported the significant correlations between AM and immune-inflammatory parameters, the SOFA score, and APACHE II score. In particular, the SOFA score showed the strongest association with the levels of mAM and tAM. Thus, the elevation of mAM and tAM levels during the perioperative period may reflect the amplified and prolonged surgical stress. The physiological roles of AM in the inflammatory response following surgical stress remain to be elucidated.
